# Transcatheter Aortic Valve Implantation Access Sites: Same Goals, Distinct Aspects, Various Merits and Demerits

**DOI:** 10.3390/jcdd11010004

**Published:** 2023-12-22

**Authors:** Odysseas Katsaros, Anastasios Apostolos, Nikolaos Ktenopoulos, Leonidas Koliastasis, Ioannis Kachrimanidis, Maria Drakopoulou, Theofanis Korovesis, Antonios Karanasos, Sotirios Tsalamandris, George Latsios, Andreas Synetos, Konstantinos Tsioufis, Konstantinos Toutouzas

**Affiliations:** 1 First Department of Cardiology, National and Kapodistrian University of Athens, Hippokration General Hospital of Athens, 11527 Athens, Greece; anastasisapostolos@gmail.com (A.A.); nikosktenop@gmail.com (N.K.); lkoliastasis@gmail.com (L.K.); iskachrimanidis@gmail.com (I.K.); mdrakopoulou@hotmail.com (M.D.); faniskorovesis@gmail.com (T.K.); akaranasos@hotmail.com (A.K.); glatsios@gmail.com (G.L.); synetos@yahoo.com (A.S.); ktsioufis@gmail.com (K.T.); 2 Department of Cardiology, University of Brussels, CHU Saint-Pierre, 1000 Brussels, Belgium; 3 Department of Cardiology, Hippokration General Hospital of Athens, 11527 Athens, Greece; stsalamandris@hotmail.com

**Keywords:** aortic stenosis, TAVI, TAVR, access site, transcarotid, axillary/subclavian, transapical, transaortic, suprasternal-brachiocephalic, transcaval

## Abstract

Transcatheter aortic valve implantation (TAVI) has been established as a safe and efficacious treatment for patients with severe symptomatic aortic stenosis (AS). Despite being initially developed and indicated for high-surgical-risk patients, it is now offered to low-risk populations based on the results of large randomized controlled trials. The most common access sites in the vast majority of patients undergoing TAVI are the common femoral arteries; however, 10–20% of the patients treated with TAVI require an alternative access route, mainly due to peripheral atherosclerotic disease or complex anatomy. Hence, to achieve successful delivery and implantation of the valve, several arterial approaches have been studied, including transcarotid (TCr), axillary/subclavian (A/Sc), transapical (TAp), transaortic (TAo), suprasternal-brachiocephalic (S-B), and transcaval (TCv). This review aims to concisely summarize the most recent literature data and current guidelines as well as evaluate the various access routes for TAVI, focusing on the indications, the various special patient groups, and the advantages and disadvantages of each technique, as well as their adverse events.

## 1. Introduction

Over the past twenty years, the definitive management of aortic valve disease has shifted from surgical methods to the transcatheter era [1]. Transcatheter aortic valve implantation (TAVI) has been established as a highly reliable alternative to surgical aortic valve replacement (SAVR) [2,3]. During the last decade, heart centers have faced a continuously increasing number of TAVI procedures that overtook, in some countries, the number of standard SAVRs [4]. The first TAVI procedure was performed in 2002 by Alain Cribier in Rouen, France [5], in a 57-year-old man with critical aortic stenosis (AS) and cardiogenic shock, and since then, the procedure has been exponentially evolving. Initially, TAVI was indicated for inoperable patients with severe aortic stenosis (AS) [6], and since then, its spectrum of coverage has been widely broadened. It has become the standard treatment option for symptomatic patients with severe AS at high risk [7,8], a valid alternative option for intermediate risk [9,10,11], and it is currently expanding in low-risk patients [12,13,14,15,16]. Of note, most recent data from long follow-ups are overcoming the concerns about the low-risk groups and demonstrating TAVI as a safe and efficacious option for all patient groups [17].

There have been substantial improvement s to the predictability, safety, and efficacy of the TAVI procedure, mainly thanks to the contemporary design of transcatheter heart valves (THV) and their delivery systems, as well as the use of novel procedural adjuncts (such as for stroke protection, rhythm monitoring, and vascular closure). The procedure is evolving in terms of its indications, device technology, and peri-operative techniques.

According to the latest guidelines, the optimal access route for TAVI is transfemoral (TF), covering about 80–90% of TAVI candidates [2]. The TF route is characterized by its minimally invasive nature compared to other methods, while the fact that the procedure can be performed under conscious sedation and local anesthesia, so that intubation and mechanical ventilation are avoided, constitutes a key advantage. Also, the further expertise and training of operators, in combination with the evolution of technology, have led to a significant reduction in major access site-related vascular adverse events, which has recently been estimated at less than 10% [18].

Although there has clearly been improvement in the technical aspects and delivery sheath diameters have been reduced for all TAVI THV systems, the remaining 10–20% of patients undergoing TAVI require alternative access routes, mainly due to peripheral vascular disease or increased levels of tortuosity of the femoral and iliac vessels, rendering them unfavorable [19,20]. The main alternative approaches for TAVI include the transapical, the transaortic, the trans-subclavian, the transcarotid, the transcaval, and the suprasternal approach. A direct comparison between the safety and efficacy of TF and alternative-route TAVI is difficult since the latter usually present with severe comorbidities, are considered at higher peri-operative risk, and are constantly underrepresented in modern clinical trials [18]. At present, the data and results for alternative approaches vary from single-center case-series to multicenter registries. Since larger studies are insufficient, the operator’s preference, the experience of the center, and individualization for patient discussion by the Heart Team are the main considerations for access choice [21]. The American College of Cardiology (ACC) expert consensus decision pathway suggests the following alternative access routes to be considered at TAVI planning: transcarotid (TCr), axillary/subclavian (A/Sc), transapical (TAp), transaortic (TAo), brachiocephalic, and transcaval (TCv) [22].

In this review, our aim is to outline the most recent data and appraise the various access routes for TAVI in terms of indications and complications, based on the current guidelines and literature.

## 2. The Main Access Sites

### 2.1. Transfemoral Access

**Figure 1:**
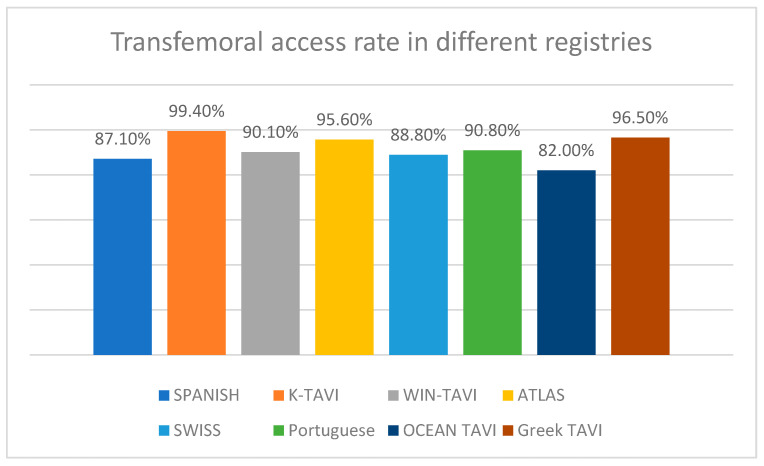
Transfemoral transcatheter aortic valve implantation (TAVI) access rates in different registries. TAVI, transcatheter aortic valve implantation.

The American guidelines suggest reconsidering surgery for patients in whom anatomy is not suitable for TF access [23], whereas the tradition of TF access is continued as it is one of the first methods that Cribier used in 2002 in the first TAVI procedures—at first he performed it transeptally with the wire across mitral valve into the aorta—and it is the most widely used vascular approach nowadays [5]. Figure 1 summarizes the TF TAVI access rates across different registries (Figure 1). However, performing TF TAVI in a patient with a suboptimal anatomy might result in not only an ineffective implementation, but it also poses an elevated risk of vascular adverse events and mortality [24,25]. As a result, the role of the heart team in reviewing all the available data is critical, including pre-operative computed tomography (CT) of the peripheral arteries and deciding the most conducive option [24].

It is important for attention to be paid, particularly during the analysis of the common femoral and iliac arteries, to their caliber (that should be at least 5.5 mm, ideally 6.5 mm for a 18F delivery system), the presence and extension of atherosclerosis and calcifications, and the degree and extension of vessel tortuosity [26]. In cases of concentric significant calcifications located anywhere from the aorto-iliac bifurcation to the femoral bifurcation, even if there is satisfactory caliber, it could potentially account for a contraindication for TF access, and then the heart team should discuss alternative routes [18]. However, in cases of ilio-femoral lesions, it is shown in the literature that ad hoc percutaneous angioplasty at this level can be combined in order to achieve a proper lumen and optimal sheath advancement, also known as the “paving and cracking technique”, a feasible, safe, and effective option to guarantee a TF approach for TAVI in such challenging anatomies. It is of paramount importance to properly assess the caliber of the iliac artery, as the optimal balloon size should be selected when transient occlusion is needed in the event of hemorrhagic complications [26]. Potential challenges such as tortuosity, the presence of aneurysms, thrombotic appositions, or extreme calcifications should definitely be identified via cautious exploration of the aorta, as all these anatomic features are possible prospective sources of peripheral embolization, reasons for vascular adverse events, and may hinder equipment delivery [2]. Other relative contraindications to TF access include large infrarenal aneurysms, with or without evidence of aortic dissection, and significant descending thoracic aortic disease [2].

On the other hand, considering the advantages of this approach, TF-TAVI is associated with minimal invasiveness, the ability to be performed without general anesthesia, facility of patient recovery, a shorter duration of hospital admissions, better clinical results, and a lower mortality rate [9,21,27,28]. This access route allows a fully percutaneous TAVI under conscious sedation or local anesthesia only. Currently, the two major commercially available THV delivery platforms are 14–16 Fr equivalent and compatible with most vessel sizes, making more than 95% of TAVR candidates suitable for the TF approach. However, despite the upgrade of device profiles and procedural techniques, TF device implementations cannot be performed in approximately 10% to 15% of patients due to iliofemoral arteriopathy, tortuosity, severe calcifications, aortic aneurysm, mural thrombus, or previous vascular surgery, with an increased risk of adverse events [29]. Access site complications include bleeding with or without hematoma emergence, dissection, pseudoaneurysm, retro-peritoneal bleeding, and acute limb ischemia. Divulged rates of access site adverse events range from 6.3 to 30.7%. Miniaturization of delivery sheath dimensions will render the majority of patients to be treated via the TF approach, decreasing incidences of vascular complications.

### 2.2. Transcarotid Access

The first TAVI via carotid access case was reported by Modine and colleagues back in 2010 [30], and two years later, they reported results from a series of 12 patients who successfully underwent TCr-TAVI using a CoreValve prosthesis [31]. The only severe adverse event that was reported was a transient ischemic accident (TIA), providing insights about the method’s safety and feasibility; however, this result is hypothesis-generating only due to the small sample size. In an in-depth review by Stonier and colleagues, they assessed the feasibility and safety of TCr-TAVI. Data from 16 studies with 72 patients—all considered to be non-eligible for a TAVI through the TF access, neither for TAp, TAo, or SC routes. The overall mortality across studies was 4.1%. There was one intraoperative death due to a rupture of the aortic annulus during balloon valvuloplasty, as well as two deaths in the 30-day post-procedure period from multi-system organ failure and hemopericardium, respectively [32,33,34]. The adverse events reported included two TIAs, the transfusion of two or more units red blood cells for 10 patients, and one acute kidney injury requiring dialysis, as well as an intraoperative dissection that was subsequently resolved [30,31,34,35,36]. The implantation of a permanent pacemaker was the most common event, with nine cases requiring one [36,37,38].

As far as the technical aspects of this access site are concerned, patient-positive candidates for this technique should have a common carotid artery diameter greater than 8 mm, with no evidence of the presence of calcification, stenosis, or severe tortuosity [39]. Furthermore, a comprehensive neurovascular workup is necessary in order to rule out the presence of severe atherosclerotic disease and also assess the patency of the anastomotic connections between the anterior and posterior circulations (circle of Willis), as the biggest hindrance of this route is the potential risk of stroke. For those who fulfil these requirements, TCr-TAVI is considered a relatively straightforward procedure [40]. In cases of occlusive events, adequate cerebral perfusion during the procedure may be ensured using passive antegrade carotid perfusion through a temporary shunt into the common carotid artery.

Limitations of the available data include the relatively small sample sizes and heterogeneity in follow-up across studies, which raise the risk of bias in not reporting the poor outcomes in early the stages of the use of this procedure [41]. However, the carotid artery is an alternative route for TAVI with good potential, and further research should be made in pursuit of support for these findings. The literature suggests that TCr-TAVI should only be considered when all other access sites are contraindicated, considering the limited experience with this procedure [39,40]. On the contrary, recent reports in experienced centers show promising outcomes. Particularly, Kirker and colleagues demonstrated that the use of TCr-TAVI in a high-volume center in the US was advantageous over other non-TF routes. More specifically, they reported that with this access site faster procedure times, fewer days of hospitalization, and comparable or even better 30-day and 1-year outcomes were achieved—comparing TCr, Tap, and TF patients—claiming that TCr-TAVI is faster and safer than TAp and has comparable characteristics to TF [42,43].

### 2.3. Axillary/Subclavian Access

It was in 2008 when Ruge and colleagues published the first-reported case of TAVI via subclavian access, and since then, numerous reports have described its use in patients not eligible for TF, Tap, or TAo approaches. There are centers that use this access route in 6 to 20% of their TAVI cases [44,45,46]. Axillary/subclavian (A/Sc) has been preferred in cases from the classical non-TF approach, thanks to being less invasive, having shorter procedure times, and the fact that general anesthesia is rarely required [45]. In this way, possible complications related to the weaning of mechanical ventilation, such as post-operative delirium, are reduced, along with the length of patients’ hospital stays [47,48]. It is a suitable and must-be-considered option for elderly, frail, or debilitated patients, and similarly to the traditional TF approach, A/Sc is an access route that has proven successful percutaneous delivery of the transcatheter heart valve, while it can also be performed via a surgical cut-down technique and also conforms to a skill that interventional cardiologists and vascular and cardiac surgeons are accustomed to [45,49,50].

Considering valve selection, balloon-expandable valves are not commonly used in this approach, with the self-expandable prostheses—such as Evolut R/PRO—being primarily used due to their smaller introducer sheaths, whereas a reasonable straight portion of the arteries would be needed for the crimped Edwards Sapien valve to be placed onto the balloon, raising the complexity of the procedure [46]. However, there are institutions, such as the one that Jarrett et al. reported, where A/Sc TAVI has become the preferred non-TF access point, even when balloon expandable valves are used [51].

Moreover, in the US CoreValve High-Risk study results, authors reported lower 30-day mortality rates for A/Sc access than with transthoracic (TAo/TAp) access (8.6% and 13.6%, respectively), presumably being less invasive, while data from the UK registry demonstrated that the A/Sc access was the only non-TF access site for TAVI, with the survival rates not significantly different from those of the TF approach, being considered as the safest non-TF access site. From this same study, survival rates were almost the same for the TAp and TAo approaches, which is noteworthy lower than post-TF or A/Sc-TAVI [52,53]. Congruous with those results are the findings from the Italian CoreValve Registry, where intra-procedural and 2-year results post-A/Sc and TF-TAVI were comparable, strengthening the belief that A/Sc access could be a valid option, even in cases where the TF route is difficult but feasible. In this study, 141 A/Sc-TAVI procedure were compared to 141 TF-TAVIs, showing that although the pre-operative risk in the A/Sc group was significantly higher, mainly due to the higher prevalence of coronary, cerebral, and peripheral artery disease, eventually there were no significant differences in procedural success and mortality [48]. From the CoreValve US Pivotal Trial and Continued Access Study, published by Gleason and colleagues in 2017, we obtain the comparison of an A/Sc-TAVI cohort of patients with a TF cohort from the same trials, with the authors finding that the results demonstrated no significant differences in outcomes, with an equivalence to TF-TAVI 30-day and 1-year mortality rates [54].

The major drawbacks of this route are vascular complications, since the subclavian artery wall is a thinner and more fragile vessel than the femoral artery [50,55]. Similarly to TF access, A/Sc access should take into consideration vessel caliber and the presence of calcification [56]. A point of huge debate is whether patients with a patent left internal mammary artery (LIMA) graft should be eligible for this site of access or not, as there are reports showing great risk of occlusion from the sheath in the subclavian artery, so accordingly, the A/Sc route should not be advisable in these patients, while others have demonstrated that it is safe if performed while employing great attention to advancing the sheath across the origin of LIMA [46,57,58]. Another point of concern found in the literature is about the feasibility of optimal axillary artery closure, as manual compression of the site of puncture is not possible, concerning mostly the percutaneous rather than the surgical cut-down approach [45]. Another possible downside of this site is the increased risk of neurological complications due to the proximity of the brachial plexus [56]. The left subclavian artery is the most favorable option for A/Sc-TAVI since the right side often has an unfavorable implantation angle, even though there are a respectable number of cases in which right subclavian access has been used with success [44,47].

As attested above, the TF approach remains the standard access site for TAVI; however, as there will still be patients who are not suitable for TF access, it has to be determined if A/Sc is the best alternative, assuming that there is emerging evidence towards the non-inferiority of the A/Sc approach.

### 2.4. Transapical Access

The first TAVI procedure through the TAp route was performed in 2005 using an Edwards Sapien valve, which was the primary alternative to the TF access point at many institutions when TF was contraindicated or unable to be performed [39,46,59,60]. The advantages of this technique include fewer vascular complications, less use of intravenous contrast media and fluoroscopy, a shorter distance from the sheath to the aortic annulus, and improved alignment of the valve prior to deployment, which reduces paravalvular leaks in comparison to TF-TAVI. The fact that the TAp route of access is not restricted by peripheral vascular anatomy and size gives this access site another important positive point, as it enables the accommodation of larger sheaths since the apex is conveniently exposed and accessible in almost every patient [39,46,61].

Moving to the disadvantages of this procedure, its invasive nature is the most serious, due to which it is claimed to be a procedure of higher risk, associated with increased morbidity and mortality, particularly concerning frail elderly patients. Tap-TAVI is relatively contraindicated in cases of low left ventricular ejection fraction (EF) and in the presence of significant parenchymal lung disease (defined as forced expiratory volume in 1 s (FEV1) <35–40% of predicted values), given the fact that general anesthesia is required and myocardial damage may occur at the access site [39,41,62]. The most critical procedure time concerns hemostatic control of the access point on the apex, with several techniques—surgical as well as new sutureless apical closure devices—being described, intending to minimize incision size and blood loss [63,64]. Data from the European SOURCE registry report a higher incidence of major bleeding among patients undergoing TAp (3.9%) than for TF (2.3%) procedures. Nevertheless, TF patients have significantly higher major (11.3% vs. 2.0%) and minor (10.4% vs. 1.0%) vascular access-related complications compared to TAp [65]. The higher risk of TAp-TAVI is conceivably due to complications such as bleeding from the puncture site, ventricular apex pseudo-aneurysm, accidental damage to a coronary artery, myocardial injury potentially contributing to arrhythmias, and new wall abnormalities on the apex long term [64,66,67,68]. We should also mention that in spite of less contrast being used in TAp-TAVI, it has been associated with a significantly higher risk of developing acute kidney injury of any grade, but the explanation might be the known association between surgical trauma, systemic responses to inflammation, and renal damage [69]. Furthermore, data from analysis of the OBSERVANT registry demonstrated that despite being performed from a direct antegrade approach, the risk of a stroke was not decreased via TAp access, something that had been proposed in certain studies [70,71,72,73].

Interestingly, we notice that the more experienced the centers on the TAp approach were, the better outcomes were demonstrated, so in that context, there is a respectful possibility of a significant volume–outcome relationship between the novel TAp technique and the well-established TF procedures [74]. About a month ago (April 2023), D’Onofrio and colleagues published their 10-year experience with TAp-TAVI. The Kaplan–Meier overall survival rates at 2 years were 75% (95% CI: 69–81), 44% at 5 years (95% CI: 36–53), and 15% at 8 years (95% CI: 8–26). The authors demonstrated that hemodynamic evaluation of all study devices showed good performance at follow-up, presenting no differences between the two currently commercially available TAVI prostheses, as well as suggesting that TAp-TAVI is an optimal alternative access point whenever the TF route is not feasible, while machine learning techniques (MLTs) represent an interesting new tool for risk prediction of survival for both the early postoperative period and during follow-up [75]. Last but not least, we could not ignore referring to the study of Kofler et al., who investigated the value of the EuroSCORE II and STS scores with regard to the prediction of 30-day and cumulative mortality according to the site of access for TAVI. The study included 607 (51%) patients who underwent TAVI via the TF route and 585 (49%) who received TAVI through the TAp site at two centers between 2008 and 2016. They found that contrary to TF TAVI, the EuroSCORE II (OR 1.038, 95% CI 1.009–1.068; *p*  =  0.010) and the STS score (OR: 1.063, 95% CI 1.025–1.102; *p*  =  0.001) were independent predictors of 30-day mortality and cumulative mortality (EuroSCORE II: HR 1.023, 95% CI 1.009–1.037; *p*  =  0.001; STS score: HR 1.055, 95% CI 1.037–1.073; *p*  <  0.001) in patients undergoing TA TAVI, leading to the conclusion that the two scores were more accurate for TAp than the TF route [76].

### 2.5. Transaortic Access

In 2009, we came across the first TAo-TAVI case, published by Bauernschmitt and colleagues [77]. Despite the more invasive nature and the variety of outcomes, the direct access to the ascending aorta provided via the TAo access appeared as a promising alternative to TF procedures [78]. Inevitably, operators used this technique with both Edwards and Medtronic valve systems [79,80]. The TAo approach has become increasingly preferred among non-TF patients, avoiding the major risks and contraindications of the TAp approach [39,78,81]. There are no significant differences in the literature concerning TAp and TAo approaches and their complications, while there are documented equivalent or higher 30-day mortality rates, as well as lower 1-year mortality rates and significantly lower cardiovascular-related mortality in TAo groups [82,83,84]. In a meta-analysis by O’Sullivan and colleagues, they used 10 studies incorporating 1736 patients that compared TAp with TAo access points, with pooled success rates of 96.3% for TAo and 93.7% for Tap. No significant differences appeared in 30-day mortality, stroke/TIA, major bleeding, high-degree atrioventricular block requiring pacemaker implantation, or paravalvular regurgitation [84].

However, there are centers where the TAo route has been selected, although patients were suitable for TAp and/or TF-TAVI. For example, half (48%) of the patients in the ROUTE registry that prospectively enrolled 301 patients undergoing TAVI at 18 centers in Europe from February 2013 to February 2015 received TAVI via TAo access as per center preference, although they were suitable for TF or TA access [85]. In comparison with the other routes, TAo access provides a more direct approach and visualization of the aorta and facilitates positioning, leading to more comfortable and better alignment and deployment, as stated by experienced operators, mostly cardiac surgeons that are more familiar with great vessels’ manipulation. Furthermore, the risk of occlusive complications related to vessel injury appears to decrease as we avoid narrower arteries, such as the iliofemoral or subclavian arteries [86].

Conversely, the primary disadvantage of the TAo approach, similar to the TA route, is its more invasive nature, especially when access to the ascending aorta through sternotomy or thoracotomy is needed [46]. Additionally, although the porcelain aorta is the primary contraindication for this type of access, when considering patients with previous sternotomy or bypass grafts overlying the aorta, careful evaluation must be taken, as for many such patients, this is a definite contraindication [39]. While data on TAo-TAVI procedures in larger series are lacking, operators’ preferences seem not to be attracted by this approach [53,66].

### 2.6. Transcaval Access

TCv, or caval-aortic access, involves delivery of the vascular sheaths into the abdominal aorta through the inferior vena cava (IVC) via the femoral vein. The procedure requires access to the abdominal aorta, which is facilitated by an electrified caval guidewire, which is advanced into a pre-positioned aortic snare and exchanged by a rigid guidewire. By the end of the procedure, TCv access ports are closed with nitinol cardiac occluders [87]. The first 19 cases were reported in 2014 from the Henry Ford Hospital (Detroit, MI, USA), and the rationale is that iliofemoral veins are wider and more compliant than their corresponding arteries, while the IVC is close to the abdominal aorta, usually without interposed structures. Furthermore, traumatic or aneurysmal aorto-caval fistulas seldom cause immediate hemodynamic compromise. In that early series, the significant reported complications were the necessity for endograft therapy and blood transfusion, which were required in 10.5% and 79%, respectively. The majority had a residual aorto-caval shunt upon discharge, not hemodynamically significant, which was occluded in 15 of 18 patients by 42 days (ranging from 7 to 189 days). Interestingly, the overall procedural time was similar to TF-TAVI, including all vascular and hemostasis maneuvers [88].

Three years later, the same team published their results after conducting a prospective, independently adjudicated, multicenter, single-arm trial of TCv-TAVI in 100 patients. This time, bleeding and vascular complications declined owing to technique refinements, such as the complete reversal of heparin anticoagulation before closure, the implantation of slightly oversized closure devices, the use of a deflectable sheath to rotate the closure device horizontally during deployment, and the unbigoted use of balloon aortic tamponade. Centers with more trans-caval experience trended towards fewer complications, and according to the authors, outcomes might be improved using a purpose-built hemostatic closure device [87]. Based on that principle, they published the results of a 12-month prospective TCv-TAVI study, in which they used off-label commercially available nitinol cardiac occluders. One year of follow-up was reassuring, with no deaths attributed to TCv access and no major vascular complications occurring after the index procedure, whereas most fistulas and other aortic abnormalities were automatically resolved [89].

Six years after the first results about the safety and efficacy of TCv-TAVI, a multicenter European registry on 50 patients treated by TCv-TAVI showed significant progress in the approach; more specifically, VARC-2-defined life-threatening bleeding and major vascular complications significantly decreased, at 4% and 10%, respectively [90]. To conclude, the same team in 2023 published a state-of-the-art review analyzing the patient selection process, CT planning, step-by-step access and closure, management of complications, and procedural troubleshooting in special situations [91].

### 2.7. Suprasternal-Brachiocephalic Access

In recent years, specifically in 2015, we came across the introduction of a contemporary technique of TAVI implantation through the brachiocephalic (also known as innominate) artery [92]. This suprasternal-brachiocephalic TAVI (S-B) route, for patients not suitable for a TF, Tap, or TAo approach, facilitates the avoidance of thoracotomy and is of great interest and utility.

The dominant contraindications for an S-B access include severe calcification of the ostium of the brachiocephalic artery, smaller size or tortuosity of the vessel, and—preventative for the patient’s passive neck extension—deformity of cervical spine anatomy. The advantages of the procedure include the safe placement of large sheaths, which leads to fewer vascular and cerebrovascular complications; the elimination of the need for aortic arch instrumentation with the valve delivery system in order to reduce embolic events; the maximal precision during valve positioning and deployment thanks to the direct placement of large sheaths in the aorta and the brachiocephalic artery; increased stability during valve delivery; and improved accuracy in deployment since the entry point of the delivery catheter is closer to the aortic valve annulus [93].

Philipsen et al. presented their results from the procedure on 20 individuals, with survival rates of 85% at 6 months and 75% at 1 year—neither deaths were related to the procedure or the valve—at a mean follow-up time of 497 ± 256 days [94]. Carpetti and colleagues studied 26 people not suitable for TF or A/Sc access and with relative contraindications for TAp route high-risk patients who underwent TAVI via the S-B approach. In 88.4% (n = 23) of the patients, the procedure was performed as intended, whereas in 11.5% (n = 3) patients, there was a conversion to right carotid access. At 30 days, they reported one major stroke (3.8%) and three access-site-related vascular complications (11.5%), with no deaths. Within a mean follow-up of 317 days (57–705), two patients were deceased—both from cardiovascular causes—while of the twenty-four survivors, nineteen (79.2%) were in NYHA functional Class I or II [95]. Codner et al. went through the S-B-TAVIs of 11 patients unsuitable for TF-TAVI. After propensity matching for baseline characteristics, they compared S-B patients to patients undergoing TAo, Tap, and A/Sc TAVI. Patients treated through S-B and A/Sc access had shorter procedure times and were able to mobilize earlier than TAo-route patients. There was a median of 1.6 days (interquartile range (IQR): 0.9–1.8) for S-B-TAVI, 1.6 days (IQR: 0.9–2.7) for A/Sc-TAVI, and 3.9 days (IQR: 1.9–4.5) for Tao-TAVI after the procedure (*p* = 0.001). The length of hospitalization was shorter for patients treated via S-B or A/Sc access compared to patients treated via Tao or Tap approaches: a median of 4 days (IQR: 3–8) for S-B and 4 days (IQR: 4 to 8) for A/Sc versus 8 days (IQR: 6 to 14) for Tao and 6 days (IQR: 7 to 11) for Tap accesses (*p* = 0.01) [96]. Later, Eudailey and colleagues retrospectively reviewed 84 patients who had undergone S-B-TAVI. All cases were characterized by technical success, and in 30 days, the patient survival rate was 98.8% (n = 83), only minor complications were reported, and—most notably—there were no TIAs or strokes (0%, n = 0). Re-exploration was 3.6% (n = 3) for bleeding and 1.7% (n = 1) for major bleeding. The mean length of stay in the intensive care unit was 1.42  ±  1.23 days, and the length of hospitalization was 4.20  ±  3.29 days [97]. Overall, S-B seems to be a feasible and safe approach for TAVI when TF access is not a possible choice.

**Figure 2:**
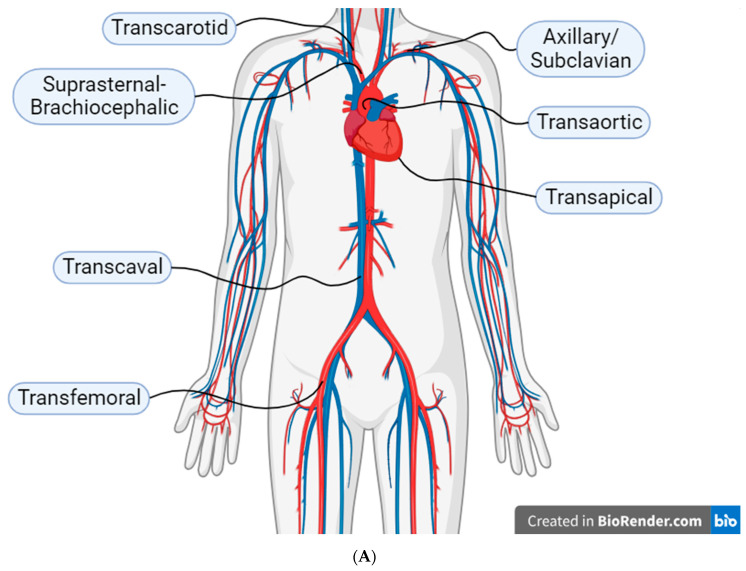

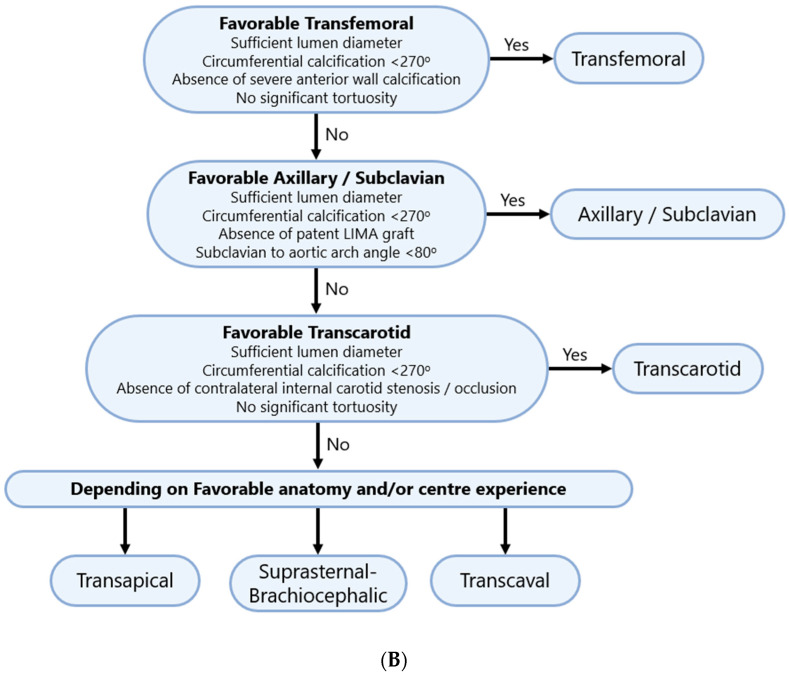
(**A**) The main transcatheter aortic valve implantation (TAVI) access sites. (**B**) Factors for deciding the appropriate vascular access for TAVI. TAVI, transcatheter aortic valve implantation; LIMA, left internal mammary artery.

Figure 2A illustrates the main TAVI access sites and (B) the factors that the heart team is proposed to take into consideration for deciding the appropriate vascular access for TAVI (Figure 2A,B).

Furthermore, Table 1 summarizes the advantages and disadvantages of the access approaches in TAVI.

## 3. Guidelines about Access Site Selection

The increased number of TAVIs performed annually on a global scale has driven the publication of consensus statements and guidelines regarding indications of TAVI and special techniques that should be followed. An expert consensus paper was published by the American College of Cardiology (ACC) in 2017 [22]. According to Otto et al., transfemoral TAVI should be the first choice when possible. They highlight that the significant reduction in sheath diameters for delivery platforms made transfemoral access feasible for the majority of patients, even for those with lower BSA. However, atherosclerotic load and location, diameter and tortuosity of arteries, and the presence of a mural thrombus should be taken into consideration prior to the selection of an access site. Moreover, they support the idea that other access sites, such as Tao, A/Sc, and TA, should be preferred as alternatives. TCr and TCv could be considered third-line solutions, but only they are when performed by operators and centers with high experience and workloads in this setting.

According to the first expert consensus statement, periprocedural vascular ultrasound is mandatory for transfemoral arterial access, and preprocedural planning is realized, mainly with computed tomography. The transfemoral approach includes both percutaneous and cut-down access, with the first being the first-choice percutaneous method, which should be preferred in arteries with larger diameters and without atherosclerotic disease or calcification, as well as in patients with wound-healing concerns. Rarely, the percutaneous approach is converted to a cut-down due to periprocedural complications, and then surgical techniques are required to ensure hemostasis and the artery’s patency. For transapical and transaortic cases, multimodality imaging and specialized surgical techniques are required to achieve optimal access [22].

The European Society of Cardiology (ESC) and the European Association of Cardiothoracic Surgeons (EACTS) published mutual guidelines of the management of valvular heart disease in 2017 [98]. The available data supported the fact that TAVI is superior regarding mortality compared to medical therapies in extreme-risk patients, non-inferior or superior to surgery in high-risk patients, and non-inferior to surgery or even superior when transfemoral access is possible in intermediate-risk patients. The authors support the fact that when TAVI is feasible via the femoral artery, it should be performed, even in lower-risk patients.

In 2018, numerous cardiological and cardiothoracic scientific societies in the United States published a consensus paper on operator and hospital suggestions and recommendations for TAVI. While this statement is not focused on technical aspects, it supports the fact that transfemoral access should be preferred over other routes. According to this, every proceduralist should have prior TAVI experience with participation in 100 transfemoral TAVI with at least 50 as the first operator. Moreover, if an institution would like to expand to other access routes, such as TAp, TAo, brachiocephalic arteries, or TCv, it should first document the completion of 80 TAVI procedures via the transfemoral approach with an STS/ACC TVT registry 30-day risk-adjusted TAVI all-cause mortality, at least “as expected” or “better than expected” [99].

ACC and AHA have extensively included TAVI in the updated guidelines about the management of valvular heart disease in 2020 [23]. First of all, transfemoral access has been considered a minimum requirement for a center to be considered a primary (level II) valve center. The authors support that the current evidence regarding the superiority or non-inferiority of TAVI versus SAVR was established when TAVI was performed through the femoral artery. The existing literature supports the fact that the mortality rate is higher with non-transfemoral TAVI than with SAVR, which is probably due to the access approach itself, and it is also likely due to the higher comorbidity burden and risk in patients with severe peripheral vascular disease that impede transfemoral access. When transfemoral TAVI is not feasible, TAVI would not be the first choice per se, but shared decision-making, including SAVR and palliative care options, is required.

ESC and EACTS revised and updated their 2017 version guidelines in 2021, empowering the role of TAVI in the management of aortic stenosis with higher-quality evidence. The suitability for transfemoral access became an important criterion for selection between TAVI and SAVR. When this criterion is fulfilled along with another one (age > 75 years old or unsuitable/high risk for SAVR (STS-PROM/EUROSCORE II > 8%), TAVI should be performed. When the transfemoral approach is impossible or challenging, SAVR feasibility plays a major role in the final decision of the heart team. In the current guidelines, the authors do not clarify which approach should be used when the transfemoral approach is unsuitable [2].

## 4. Trends—What Is Happening Now

Due to experience and feasibility, TF access is the preferred route in most clinical trials, and it is advocated as the first option by all guidelines and consensus documents. With the reduction in delivery system and sheath dimensions, the majority of TAVI candidates can be treated via the TF route, and the incidence of vascular adverse events has decreased. However, concomitant severe peripheral artery disease is frequent in this population and increases the risk of vascular complications. For patients with severely calcific/stenotic iliofemoral arteries, pre-TAVI balloon or/and stent angioplasty as well as intravascular lithotripsy, have more recently been shown to be feasible and to facilitate TF large-bore arterial access [100].

Once the TF approach is deemed unsuitable, the choice between TA, A/Sc, (TAo)/direct aortic (DA), and other alternative access (TCr, or TCv) will largely depend upon the presence of comorbid conditions along with operator and institutional experience. Each access option has unique advantages and restraints and must be carefully assessed in the context of a patient’s anatomy, applying a multidisciplinary approach in which the heart team plays a key role. The access site is critical not only to improve the feasibility of the procedure, but also to improve outcomes, as it is directly linked to possible vascular and bleeding adverse events. Every heart team should be able to offer more than one non-TF option to individualize the treatment for every single inoperable individual with severe AS.

## 5. The Secondary Access in TAVI

In every TAVI procedure, there are two access sites utilized. The primary—analyzed above—as well as a secondary access site, which is used for the introduction of catheters for angiography, aiding device placement in order to obtain invasive hemodynamic data [101]. There are two main sites used for secondary access in TAVI: the contralateral femoral artery and the radial artery. Routinely, the transfemoral site is the preferred secondary access site; however, radial access—a well-known alternative to femoral access in coronary procedures—is associated with a reduction in vascular and bleeding events and mortality [102,103], so similarly to the coronary field, the results of a handful of studies directly comparing outcomes in transfemoral TF and transradial secondary access sites in TAVI showed a major reduction in vascular and bleeding complications and improved 30-day outcomes associated with the use of the transradial approach as the secondary TAVI site of access [101,104,105,106,107,108]. To take this further, when there are such promising and favorable results with the transradial secondary TAVI access site, there should be similar, if not better, results with the distal radial artery. Studies by Achim et al. demonstrated its feasibility and potential benefits without compromising the safety of the procedure. The fewer arterial obstruction complications and the shorter duration to succeed hemostasis are key advantages of this technique. However, larger randomized trials are needed for further evaluation [109,110,111].

## 6. Discussion—Conclusions

There is no doubt that the TF route has established itself over those past years where TAVI has been performed as the first choice of access, especially moving to the era of new minimalistic procedures for more patients in intermediate and low-risk groups. At the time that this review is being written, there are no official and definite recommendations, so TAVI operators facing difficulties with the TF route rely on local expertise and thorough heart team analyses of each individual patient’s characteristics in order to select and design the most appropriate access route. In that context, selecting the optimal access site requires individualization and careful cogitation of the patient’s specific anatomy as well as of the specific THV that fulfills those unique requirements and matches them best. Reviewing the relevant literature, it is evident that the success and better results of each specific access site, apart from the patient’s suitability, depend heavily on the expertise of the operator and the operating team. There is definitely a learning curve, varying according to the complexity of the type of access route and the familiarity of the operator. Taking all of the above into serious consideration and order, it can give each center a higher level of confidence and comfort with the alternative access approaches and the readiness to individualize every decision, thus elevating the rate of successful TAVI procedures. Someone could not just ask for guidelines without previously suggesting and emphasizing the need for further randomized studies with larger cohorts and long-term follow-ups thoroughly investigating the impact of the alternative approaches on short- and long-term outcomes.

## Figures and Tables

**Table 1:** Advantages and disadvantages of the access approaches in TAVI.

Access Route	Advantages	Disadvantages
Transfemoral	The most widely used vascular approach nowadays. Lower invasiveness. Ability to be performed without general anesthesia. Ease of patient recovery. Shorter hospital stays. Better clinical outcomes and a lower mortality rate. Allows a fully percutaneous TAVI ^1^ under conscious sedation or even local anesthesia only.	Vascular restrictions and contraindications such as the presence and extension of atherosclerotic plaques, calcifications, and degree and extension of tortuosity.
Transcarotid	Percutaneous option, less invasive than transthoracic access. Straightforward procedure with many similarities to carotid endarterectomy.	Potential higher risk of stroke requiring comprehensive neurovascular workup to rule out significant atherosclerotic disease and patency assessment of the Circle of Willis. Sparse follow-up data.
Axillary/subclavian	Percutaneous technique. Less invasive than transthoracic accesses. Shorter procedure time and length of hospital stay. Decreased need for general anesthesia. Procedure similar to TF ^2^ approach and A/Sc ^3^ access is familiar to cardiac surgeons.	Thinner and more frail than femoral artery vessels that elevate the risk of vessel injury compared to TF ^2^ access. Restrictions concerning vessel caliber and presence of calcification—as in TF ^2^. Care must be taken in patients with previous CABG ^4^ procedures involving LIMA ^5^ graft in cases of occlusion if crossing LIMA ^5^ origin. Most cases require a cut-down closure approach of the axillary artery. Risk of brachial plexus injury due to the proximity of the vessel to the brachial plexus.
Transapical	Fewer vascular complications. Fewer contrasts and fluoroscopy used. Short distance from sheath to annulus that leads to improved valve alignment before deployment and fewer PVLs ^6^. Enables the accommodation of larger sheaths.	More invasive technique with a risk of myocardial injury. Complications related to puncture site (bleeding, ventricular apex pseudo-aneurysm, and accidental coronary artery damage) resulting in arrhythmias or wall motion abnormalities. Relative contraindication for impaired LV systolic function (LVEF ^7^ <15–20%) and respiratory tract disease (FEV1 ^8^ <35%).
Transaortic	Fewer vascular complications. The direct visualization of the aorta facilitates valve positioning and deployment and permits rapid cannulation and initiation of cardiopulmonary bypass in the case of emergency conversion to open surgery. Familiarity of trained operators for accessing and cannulating aorta. Less bleeding risk compared with TA ^9^ TAVI ^1^.	Invasive access technique requiring sternotomy or right mini thoracotomy. Dreaded for patients with prior sternotomy or bypass grafts overlying aorta. Contraindicated in porcelain or diseased aorta, prior CABG ^4^ with patent LIMA ^5^ and/or vein grafts with higher origin and in patients with anatomic variations that prevent optimal coaxial prosthesis deployment (i.e., pectus excavatum).
Transcaval	Femoral vein is not subject to the same limitations as the artery (such as the presence of calcification). Last percutaneous option for non-eligible patients for any other vascular access patients.	Increased risk of bleeding regarding the venous-aortic closure and short- and long-term hemostasis caval-aortic. More studies are required to establish safety and efficacy, indications, and contraindications.
Suprasternal-Brachiocephalic	Percutaneous approach. Safe placement of large sheaths with reduced vascular and cerebrovascular complications. Elimination of the need for aortic arch instrumentation with the valve delivery system in order to reduce embolic events. Maximal precision during valve positioning and deployment thanks to the direct placement of large sheaths in the aorta and the brachiocephalic artery. Increased stability during valve delivery and improved accuracy in deployment since the entry point of the delivery catheter is closer to the aortic valve annulus.	Possibility of complications similar to mediastinoscopy, such as pneumothorax, wound infection, or tracheal injury. In cases of a short ascending aorta and a too short distance between catheter entrance point and aortic annulus complete deployment of the valve outside the sheath can be hindered. Currently utilized 18-Fr delivery sheaths are often longer and therefore less ideal for central arterial access.

^1^ TAVI, transcatheter aortic valve implantation; ^2^ TF, transfemoral; ^3^ A/Sc, axillary/subclavian; ^4^ CABG, coronary artery bypass graft; ^5^ LIMA, left internal mammary artery; ^6^ PVL, paravalvular leak; ^7^ LVEF, left ventricular ejection fraction; ^8^ FEV1, force expiratory volume in 1 min; ^9^ TA, transapical.

## Data Availability

Publicly available datasets were analyzed in this study.
